# Supplementary material for: Chromothripsis during telomere crisis is independent of NHEJ, and consistent with a replicative origin
Source: Genome Res. 2019 May;29(5):737–49. doi: 10.1101/gr.240705.118 (PMC6499312; doi:10.1101/gr.240705.118)
Supplement: Supplemental Material [file supp_gr.240705.118_Supplemental_file_1.zip › contigs/annotated_contigs/DB80/contig.2.DB80_length_694_mean_cov_34.6772334294.docx]

**DB80_length_694_mean_cov_34.6772334294**

```
GGGGGGCGGGGGGGGGGGGGGGGGGGGGGGGGGGGGGGGGGGGGGGGG|GGGGGGGGGGGGG|TGGTGGGGGGGAAACCCACCTAACCG
 >chr2:33141583-33141644 + E=6e-19 p=0e+00       >chrUn_gl000220:134011-134657 - E=0e+0
ACTCACTAAATTAAGGTGAAGGGACGTGGGTAGGGGGGGGAGCCGGGGGGCAACTTGAAAATTAAACTGACCCTTCCCAAAGCCCAAGT
0
AGAAGAGTCTAGGCGCCAAAACACAAAGAAAAGTAAAGCGCCGATCAAAGAACAATAGGGCCCCCGCCAGGGCGGAGGTTCCCTAGGCG

AGGTTCCCTAGGCAACGAGGGAGAGAGGGAGGGGCCTCCAGAAGGGAGAGAGAGAAACCCGTTGCCCCAGGCTCGGTGAAGTCGGCGAG

ACCTCCCTCCGTGTCACGTCGACTTTCAATAACAGTGGCCGCTAGGTGATGCCCGAAGACAACCGATGCCTGCCTGCAAATGTCCGTCA

GCAGGGAAAAGAATTAATGAATTAATTAATTTCCGTATTTATTTAGAGACCGAGTCTCACTCACTCTACAGCCTGGGCCGTAGTGCAGT

GGCGCGATCTCGGCTCCCTGCAGCCTCCGCTTCCCTGGTTCAAGCGATTCTCCCGCCTCAGCCTCCCGAGGAGCTGGCATTACAGGGGC

CTGCCCCACCGCTCCCGACTCAGCTTTGTATTTTTAGTAGAGACGGGGTTTCGCCGTGTTGCGTCCGGCCTTA
```
